# Effects of Feeding Systems on the Growth Performance, Carcass Characteristics, and Meat Quality in Sheep: A Meta-Analysis

**DOI:** 10.3390/ani14182738

**Published:** 2024-09-21

**Authors:** Wenjie Wang, Xiaoan Zhang, Huiqing Wei, Sunze Wang, Yang Ye, Li He, Kefan Zhang, Yuan Lu, Zijun Zhang, Yafeng Huang

**Affiliations:** 1 College of Animal Science and Technology, Anhui Agricultural University, Hefei 230036, China; wangwenjie0123@stu.ahau.edu.cn (W.W.); zhangxiaoan@stu.ahau.edu.cn (X.Z.); weihuiqing123@stu.ahau.edu.cn (H.W.); wangsunze@stu.ahau.edu.cn (S.W.); yeyang21@stu.ahau.edu.cn (Y.Y.); zhangkefan@stu.ahau.edu.cn (K.Z.); luyuan6868@stu.ahau.edu.cn (Y.L.); zhangzijun@ahau.edu.cn (Z.Z.); 2 Center of Agriculture Technology Cooperation and Promotion of Dingyuan County, Chuzhou 233200, China; 3 New Rural Development Research Institute, Anhui Agricultural University, Hefei 230036, China; heli@ahau.edu.cn

**Keywords:** pasture-grazing, carcass assessment, quality traits, ruminants

## Abstract

**Simple Summary:**

Demand for high-quality sheep meat with good nutritional properties and health value is increasing. We performed a meta-analysis to review how different feeding regimes affect sheep growth performance, carcass traits, and meat quality. Compared with stall-finished feeding, animals fed solely on pasture had lower carcass yield, intramuscular fat, and meat edible quality, but higher meat protein content. Pasture-fed sheep for which the diet was supplemented had comparable carcass attributes to stall-fed sheep, but improved color and meat quality.

**Abstract:**

Meat quality is important in the meat-production chain. Conflicting reports of the effects of feeding systems on sheep growth performance and meat quality exist. By way of meta-analysis, we reviewed the literature on the growth and slaughter performance, and meat quality of lambs that grazed solely on pasture, those that grazed on pasture but received a dietary supplement, and those were exclusively fed indoors. The relevant literature comprised 28 papers, from which response variables of interest were obtained. Compared with stall-fed sheep, pasture-grazing led to significantly (*p* < 0.05) lower average daily gain, slaughter live weight, hot carcass weight, cold carcass weight, and similar dressing percentage, but pasture-grazed sheep fed a supplement had similar (*p* > 0.05) values for each of these attributes to stall-fed sheep. The quality of the longissimus muscle from lambs that grazed either exclusively on pasture or pasture with a supplement had significantly (*p* < 0.05) lower lightness and intramuscular fat content, and significantly (*p* < 0.05) higher yellowness, Warner–Bratzler shear force, and protein content than meat from stall-fed sheep. We conclude that sheep that have fed exclusively on pasture have lower carcass yield and meat edibility, but improved meat quality, and that pasture-fed sheep that received a supplement had comparable carcass attributes, but greater meat color and health quality than stall-fed sheep.

## 1. Introduction

Increased demand for high-quality meat with good nutritional properties and health value presents a number of challenges for the sheep industry [1,2]. Evidence of the nutritional value of lamb meat is needed to promote its inclusion in diets, particularly for infants [3] and the elderly [4]. To satisfy the increased demand for high quality, both the production and improvement of lamb meat quality must be improved. The growth rate, carcass and quality attributes of sheep meat are correlated with production factors in the farming system such as breed/sex, agroclimatic conditions, socio-economic environment, and management system [5,6,7], of which feeding regimen seems to be one of the most important environmental factors [8].

The feeding systems that are commonly used in sheep farming include pasture/grassland grazing, indoor-fed/concentrate-fed, and pasture-grazing augmented with some dietary supplement [9]. Compared with indoor-fed lambs, pasture-grazing lambs have lower average daily gain (ADG), slaughter live weight (SLW), hot carcass weight (HCW), cold carcass weight (CCW), and dressing percentage (DP) but a lower intramuscular fat (IMF) content (considered to be healthy for human consumption), while supplemented-grazing lambs had comparable SLW, HCW, CCW, DP and color, tenderness, and juiciness of lamb meat than pasture-grazing [10,11]. Conversely, Wang et al. [12] found that pasture-grazing sheep have similar ADG and HCW, but lower IMF content than indoor feeding sheep, while pasture-grazing lambs with the concentrate supplemented increased the ADG and HCW but had no change IMF content than compared with indoor-fed ones. In addition, pasture-grazing lambs with a lucerne-supplemented diet have increased ADG and SLW and improved tenderness, juiciness, fat degree, and meat flavor [13]. However, SLW, HCW, CCW, DP and color, tenderness, and juiciness of lamb meat are comparable between the concentrate-fed, and grazing Brachiaria pastures supplemented with concentrate [10]. Therefore, investigations in published reports have demonstrated conflicting results in the growth performance, carcass characteristics, and meat quality in sheep. Furthermore, the results from single experiments may not necessarily provide a definitive understanding of how feeding systems affect lamb productivity, carcass characteristics, and meat quality.

While previous published reviews of Huang et al. [7] or Ke et al. [9] examined the effects of feeding systems on sheep growth rate and carcass and meat quality attributes, these reviews are based on a synthesis of results from individual studies, based on the results of narratives with subjective and qualitative summaries, or where few quantitative methods have been applied to compare pasture-grazed and indoor-fed lambs using a single feature quality. According to several authors [14,15], a meta-analysis approach quantitatively analyzing the results of individual studies published on the same topic with contradictory findings would obtain greater precision conclusions. To our knowledge, the effects of three feeding systems on the lambs’ growth performance, carcass traits, and meat quality have not been evaluated by a meta-analysis. Given the above, we hypothesized that 1) pasture-grazing lambs have reduced growth performance and carcass traits but improved meat quality than indoor-feeding lambs; 2) supplemented grazing lambs have comparable performance and carcass and meat quality attributes to their indoor-feeding counterparts. Consequently, the aim of this study was to examine how different feeding systems (indoor-feeding x pasture-grazing and indoor-feeding x pasture-grazing augmented with a supplement, respectively) affect sheep growth rate and carcass and meat quality attributes using a meta-analytical approach in order to identify the optimum effective feeding system for improving production efficiency and lamb meat quality.

## 2. Materials and Methods

### 2.1. Study Search Strategy

The meta-analysis was performed in agreement with the Preferred Reporting Items for Systematic Reviews (PRISMA) [16]. Search terms were defined based on principles of the PICOS strategy (Table 1, [14]) (population, intervention, comparison, and outcome). The search was performed on published article titles and abstracts (January 2003 to August 2024) in the Web of Science, Taylor & Francis Online, ScienceDirect, and PubMed databases using on same search terms. Features of included studies and extracted outcome indicators were analyzed independently by two reviewers.

### 2.2. Study Selection and Exclusion Criteria

The inclusion criteria required were as follows: papers investigating the influence of feeding systems on lamb growth rate, and carcass and meat quality attributes of the longissimus muscle. The exclusion criteria included the following: (1) papers were editorials, letters or reviews; (2) studies were duplicated or contained overlapping data; (3) studies contained duplicated or incomplete original data; and (4) studies that measured and reported data on adult sheep.

### 2.3. Data Extraction and Quality Assessment

A customized data extraction form was developed to record publication details (author, and country), methods (pasture-grazing, pasture-grazing with a supplement, and stall-fed), objects (species/cultivar, number), and evaluated variables (growth rate, carcass attributes, meat quality). Response variables included growth rate (ADG), carcass attributes (SLW, HCW, CCW, DP, and rib eye area (REA)), edible quality of the longissimus muscle (lightness (L*), redness (a*), yellowness (b*), Warner–Bratzler shear force (WBSF), water-holding capacity (WHC), cooking loss (CL), and drip loss (DL)), and nutritional quality attributes (moisture, protein, IMF, and ash). All variables were converted into the same measurement unit. We employed the Cochrane Quality Assessment Tool in combination with the enhanced key assessment tool (high-quality item rating scale-RRB) developed by Munn et al. to modify the systematic review approach for this study and assess the risk of bias in the included studies [17]. Bias assessment was carried out using RevMan 5.3 [18]. The following six criteria were examined and rated as follows: 2 for yes, 1 for indeterminate, or 0 for no. (1) Were the research objectives and problems clearly articulated? (2) Were the traits of the experimental animals explicitly defined? (3) Was the study design a randomized controlled trial? (4) Was the test outcome data complete? (5) Did the survey results show any signs of subjective influence? Studies with a total quality assessment score ≤ 6 were excluded from statistical analysis. Descriptive statistics for all parameters were collated using Microsoft Excel^®^ (version 2013) software. Data extraction and critical evaluation of article quality were conducted independently by two authors; consensus was reached in the event of any dispute.

### 2.4. Statistical Analysis—Meta-Analysis

Funnel plots were utilized to evaluate the presence of publication bias within the studies that were included. The funnel plot exhibited significant asymmetry (with studies represented by dots), indicating notable publication bias in the included studies. Egger’s test is commonly employed for assessing potential publication bias in meta-analyses through the observation of funnel plot asymmetry. A *p*-value ≥ 0.05 suggests a minimal risk of publication bias, while a *p* value < 0.05 indicates potential publication bias [19]. All data were analyzed using the STATA meta-analysis software (version 14.0; Stata Corporation, College Station, TX, USA). Calculations were based on the standardized mean difference (SMD) at a 95% confidence interval of Hedges’ g, where the mean value of the control group is X_C_ and the treatment group mean is X_E_. The calculation used the following formula [20]: SMD = (X_E_ − X_C_)/S_P_, where S_P_ is the pooled SD. The analyzed studies were weighted by the inverse of the variance for a random effect analysis using the methods of Der-Simonianand et al. [21]. Heterogeneity of results among the trials was quantified using the I^2^ index. The I^2^ is calculated using the following formula [20]: I^2^ = [Q − (K − 1)]/Q × 100, where Q is the χ^2^ heterogeneity statistic and K is the number of trials. An I^2^ value I^2^ ≤ 25% was considered as low heterogeneity; I^2^ of 26% to 75% represent moderate heterogeneity; and I^2^ > 75% represent high heterogeneity [22]. Forest plots were constructed to evaluate the effects of different systems on the ADG of lambs. Statistical significance was set to *p* < 0.05.

## 3. Results

### 3.1. Article Search and Study Characteristics

**Figure 1:**
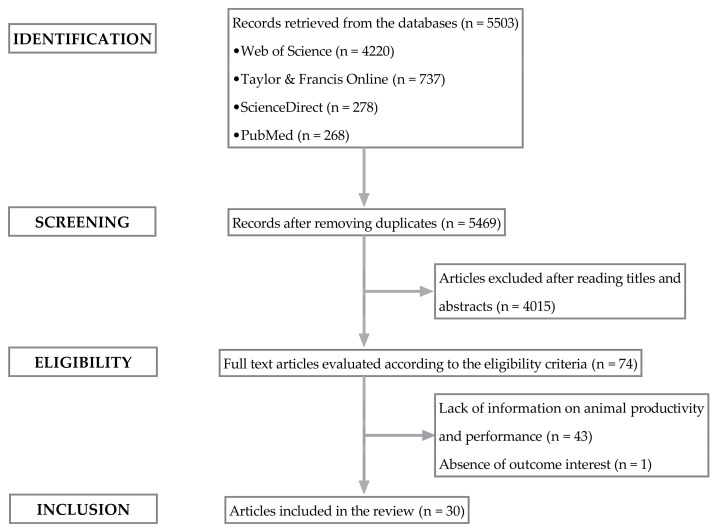
Flowchart with the selection process of the final sample of sheep lambs for the Systematic Review.

A methodological flowchart is presented in Figure 1. Of 5503 initially identified articles, 4220 were sourced from the Web of Science, 737 from Taylor & Francis Online, 278 from ScienceDirect, and 268 from PubMed. Duplicate studies, meta-analyses, reviews, conference papers, and studies in which the title and/or abstract were inconsistent with the research content were excluded. After preliminary screening, 73 studies remained. Of these, 30 studies were retained for quantitative synthesis (others lacked animal productivity and production performance data); 11 papers comparing stall-feeding to pasture-grazing, 7 comparing stall-feeding to pasture-grazing augmented with a supplement, and 12 comparing each of stall-feeding, pasture-grazing, and pasture-grazing augmented with a supplement. Outcome variables from these papers are summarized in Table 2, and descriptive statistics used in meta-analysis in Table 3 and Table 4.

### 3.2. Quality Assessment and Data Extraction

**Figure 2:**
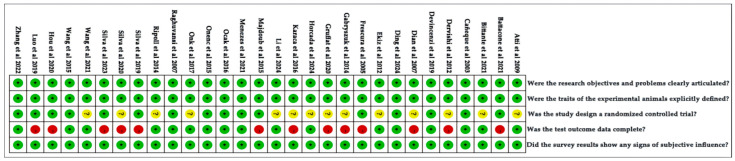
Quality evaluated of all studies screening on feeding system [8,10,12,22,23,24,25,26,27,28,29,30,31,32,33,34,35,36,37,38,39,40,41,42,43,44,46,47,48,49]. Green circles indicate the score of 2; yellow circles indicate the score of 1; red circles indicate the score of 0.

The quality of each included study was assessed and the corresponding score is illustrated in Figure 2. A score of 2 in green indicates low risk, a score of 1 in yellow suggests uncertainty, and a score of 0 in red signifies high risk. The maximum attainable score was 10 based on five quality assessment criteria, with scores ranging from 7 to 10 for the included studies. The average total quality score was 8.73 ± 0.21, with a median of 9.

### 3.3. Growth Performance

**Figure 3:**
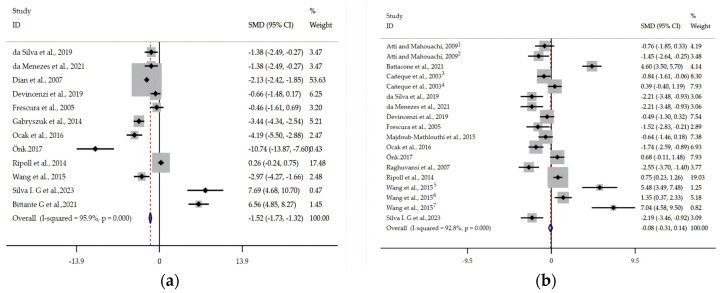
Forest plot of the effect of feeding system on average daily gain in lambs based on standardized mean differences (Std. diff in means). The lower polygon indicates the mean effect size, calculated according to a random-effects model. Square size indicates the weight of each published study relative to the mean effect size (smaller squares represent less weight). Horizontal bars represent 95% confidence intervals. (a) Stall-fed vs. pasture-grazed lambs [12,25,27,28,29,31,32,33,34,37,39,42]; (**b**) stall-fed vs. pasture-grazed lambs fed a supplement [12,23,24,26,27,28,31,32,33,36,37,39,41,42]. ^1^ Pasture-grazed lambs receiving faba bean [23]; ^2^ pasture-grazed lambs receiving soya bean [23]; ^3^ pasture-grazed lambs receiving a commercial concentrate [26]; ^4^ pasture-grazed lambs receiving whole barley with a commercial protein supplement [26]; ^5^ pasture-grazed for 2 h lambs receiving a concentrate [12]; ^6^ pasture-grazed for 4 h lambs receiving a concentrate [12]; ^7^ pasture-grazed for 8 h lambs receiving a concentrate [12].

Meta-analysis results of ADG according to Cohen’s methodology are depicted in Figure 2. Compared with meat from stall-fed lambs, pasture-grazed lambs had significantly lower ADG (SMD = −1.52; *p* < 0.001). The ADG of meat from pasture-grazed lambs fed a supplement was similar (SMD = −0.08; *p* = 0.460) to that of stall-fed lambs. Heterogeneity was significant for ADG. An Egger’s regression test found no publication bias for ADG (*p* > 0.1; Figure 3a,b).

### 3.4. Carcass Attributes

**Figure 4:**
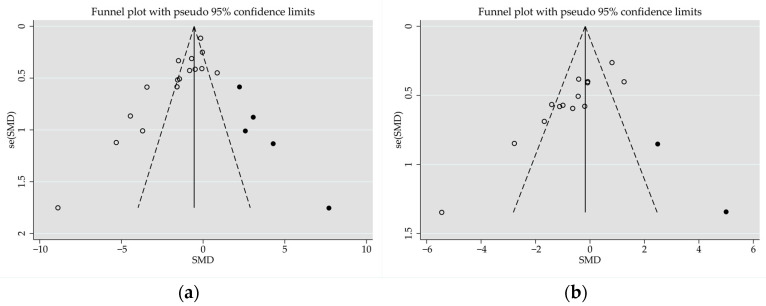
The funnel plot illustrates the standardized mean difference (Std. diff in means) of studies, with empty circles denoting: (**a**) slaughter live weight of stall-fed and pasture-grazed lambs; and (**b**) cold carcass weight of stall-fed lambs, and pasture-grazed lambs fed a supplement. Potentially missing studies are represented by solid circles imputed using the trim-and-fill method. Open diamonds illustrate the mean and confidence intervals for screening studies, whereas solid diamonds illustrate the mean and confidence intervals for theoretically imputed studies in the meta-analysis.

Effect size, heterogeneity, and publication bias for the effect of feeding system on carcass attributes are detailed Table 5. Compared with stall-fed lambs, pasture-grazed lambs had significantly lower SLW (SMD = −0.59; *p* < 0.001), HCW (SMD = −0.41; *p* = 0.003), CCW (SMD = −0.68; *p* < 0.001), and REA (SMD = −1.57; *p* < 0.001), but there was no significant difference in DP (SMD = −0.13; *p* = 0.387). Heterogeneity was significant for carcass attributes between stall-fed and pasture-grazed lambs. An Egger’s regression test showed the publication bias for SLW (*p* < 0.001; four missing observations to the right of the funnel plots), while no publication bias was detected for other carcass attributes (Figure 4a).

Lambs grazed on pasture and fed supplements had similar SLW (SMD = 0.06; *p* = 0.588), HCW (SMD = 0.08; *p* = 0.578), CCW (SMD = −0.17; *p* = 0.159), DP (SMD = 0.15; *p* = 0.201), and REA (SMD = 0.42; *p* = 0.065) to stall-fed lambs. Heterogeneity was significant for carcass attributes between these two feeding regimes (*I*^2^, Table 3). An Egger’s regression test revealed publication bias for CCW (*p* = 0.001; two missing observations to the right of the funnel plots), but there was no publication bias for other carcass attributes (Figure 4b).

### 3.5. Edible Quality Attributes of Longissimus Muscle

Effect size, heterogeneity, and publication bias for the effects of feeding system on edible quality attributes of the longissimus muscle of lambs are presented in Table 6. In color, the longissimus muscle of pasture-grazed lambs has a lower L* (SMD = −0.40; *p* = 0.014), higher b* (SMD = 0.66; *p* < 0.001) and similar a* (SMD = 0.10; *p* = 0.500) values to stall-fed lambs. Compared with stall-fed lambs, this muscle in pasture-grazed lambs that were also fed a supplement was lower L* (SMD = −0.57; *p* < 0.001), and higher a* (SMD = 0.70; *p* < 0.001) and b* (SMD = 0.47; *p* < 0.001) values were higher. Heterogeneity was significant for color (*I*^2^, Table 6). An Egger’s regression test revealed no publication bias for meat color.

Compared with stall-fed lambs, the longissimus muscle of pasture-grazed lambs had higher WBSF (SMD = 0.94; *p* < 0.001) and similar CL (SMD = 0.26; *p* = 0.244) and DL (SMD = 0.18; *p* = 0.420) values. This muscle in lambs grazed on pasture but also fed a supplement had higher WBSF (SMD = 0.44; *p* < 0.001) and CL (SMD = 0.74; *p* < 0.001), and similar DL (SMD = 0.49; *p* = 0.111) to stall-fed lambs. Heterogeneity was significant for WBSF, CL and DL (*I*^2^, Table 6). An Egger’s regression test revealed no publication bias for edible quality attributes.

### 3.6. Nutritional Quality Attributes of Longissimus Muscle

**Figure 5:**
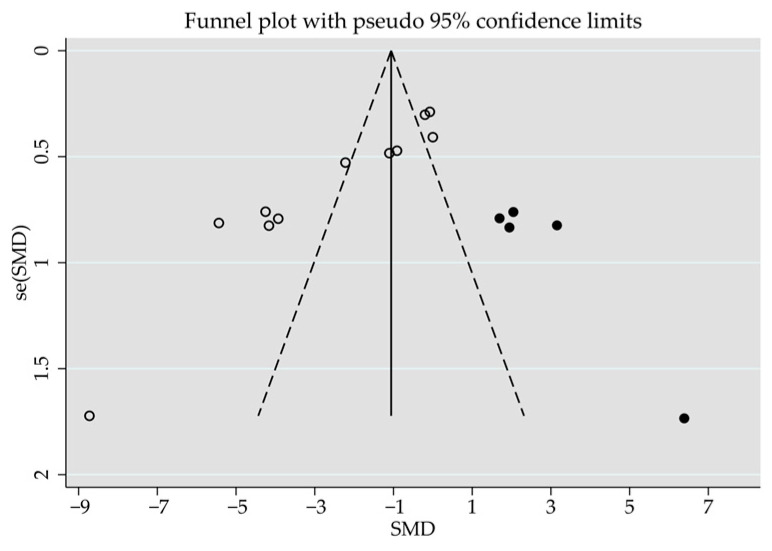
Funnel plot illustrates the standardized mean difference (Std. diff in means) of studies, represented by empty circles, comparing intramuscular fat content between stall-feeding and pasture-grazing. Potentially missing studies are represented by solid circles imputed using the trim-and-fill method. Open diamonds illustrate the mean and confidence intervals for screening studies, whereas solid diamonds illustrate the mean and confidence intervals for theoretically imputed studies in the meta-analysis.

Effect size, heterogeneity, and publication bias for the effects of feeding system on nutritional quality attributes of the longissimus muscle of lambs are detailed in Table 7. This muscle from pasture-grazed lambs had higher moisture (SMD = 0.37; *p* = 0.031), similar protein (SMD = 0.17; *p* = 0.392), but lower IMF (SMD = −0.79; *p* < 0.001) and ash (SMD = −0.80; *p* < 0.001) contents to stall-fed lambs. Heterogeneity was significant for protein and IMF between stall-fed and pasture-grazed lambs (*I*^2^, Table 7). An Egger’s regression test revealed a publication bias for IMF (*p* < 0.001; five missing observations to the right of the funnel plots), but not for other nutritional quality attributes (Figure 5).

**Figure 6:**
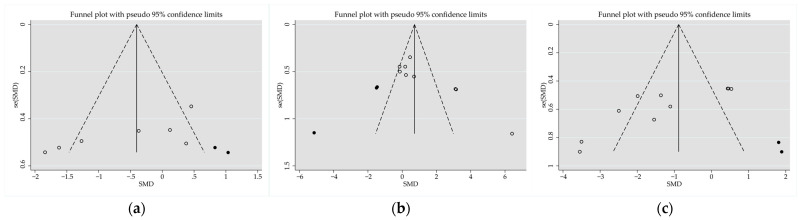
Funnel plot illustrates the standardized mean difference (Std. diff in means) of studies, represented by empty circles, comparing moisture (**a**), protein (**b**), and intramuscular fat (**c**) contents between stall-feeding and grazing pasture with supplementation. Potentially missing studies are represented by solid circles imputed using the trim-and-fill method. Open diamonds illustrate the mean and confidence intervals for screening studies, whereas solid diamonds illustrate the mean and confidence intervals for theoretically imputed studies in the meta-analysis.

The longissimus muscle of pasture-grazed lambs fed a supplemental diet had higher protein (SMD = 0.72; *p* < 0.001) and lower moisture (SMD = −0.40; *p* = 0.021), IMF (SMD = −0.88; *p* < 0.001), and ash (SMD = −0.77; *p* < 0.001) contents than stall-fed lambs. Heterogeneity was significant for nutritional quality attributes between stall-fed and pasture-grazed lambs that also received a supplement. An Egger’s regression test revealed a publication bias for moisture (*p* = 0.035; two missing observations to the right of the funnel plots), protein (*p* = 0.009; two missing observations to the left of the funnel plots) and IMF (*p* = 0.012; two missing observations to the right of the funnel plots), but not for other nutritional quality attributes (Figure 6a–c).

## 4. Discussion

Feeding system plays a crucial role in influencing the growth rate, carcass, and quality attributes of sheep meat. We conducted a meta-analysis of 31 articles from 11 countries, revealing that pasture-grazed lambs reduced growth rate, carcass attributes, and edible quality, but increased meat protein content compared to stall-fed counterparts. Additionally, supplemental grazing for lambs improved meat color and protein content but led to reduced meat tenderness. The growth performance and carcass attributes were comparable to those of stall-fed counterparts.

### 4.1. Growth Rate 

Our meta-analysis reveals that pasture-grazed lambs have lower ADG than stall-fed lambs, possibly because of the higher digestibility of concentrated diets [50], and because pasture-fed animals consume more energy by walking more (they had more space) [47] and experienced more diverse environmental conditions in pastures. Pasture-grazing can reduce ruminal bacteria *Proteobacteria* and *Fibrobacteres* [51], which play important roles in cellulose degradation [52,53], partly explaining the lower ADG in pasture-grazed sheep muscle. Another review also concluded that pasture-grazed lambs had lower ADG than their stall-fed counterparts [9].

A similar ADG was reported for pasture-grazed lambs fed a supplement and stall-fed lambs, suggesting that concentrated supplements accelerate the growth rate of grazed lambs. These effects could be due, at least in part, to the higher energy intake and optimal balance of energy, protein mineral in concentrates of lambs grazing pasture plus supplementation. Broadly consistent with our findings, a separate review [9] also reached the conclusion that lambs reared on supplementary grazing demonstrate comparable or superior ADG in comparison to stall-fed lambs. Supplementation increases energy supply and promotes growth. Chikwanha et al. [54] and Wang et al. [55] observed that as the dietary nutrition or energy level increased, a significant improve growth performance was observed in lambs. In addition, fecal Firmicutes, *RuminococcaceaeUCG-005*, and *RuminococcaceaeUCG-010*, mostly involved in decomposition and metabolism of fiber, were all increased in pasture-grazed lambs fed a supplemental diet [53]. These effects partly explain the lack of significant differences in ADG values of stall-fed lambs compared with those grazed on pasture and fed a supplement [53,56]. Ke et al. [9] also reported a lower ADG in lambs that only grazed pasture compared with stall-fed animals, and pasture-fed lambs that had been fed a supplement.

### 4.2. Slaughter Performance

Carcass quality, an important indicator of the marketable attributes of meat (e.g., meat per animal), directly affects sheep farmer income [57,58]). Pasture-grazed lambs had lower SLW, HCW, CCW, and REA than stall-fed animals, indicating that stall-fed fattening increased meat production. Another meta-analysis [59] also concluded that pasture-grazed lambs had lower SLW, HCW, CCW, and REA than stall-fed lambs. This effect is not unexpected, because sheep fed pasture had a lower ADG—a parameter that is generally positively related to sheep carcass quality.

We identified no significant difference in the carcasses of lambs grazed on pasture augmented with a supplement to those fed in stalls, suggesting that pasture-grazing and a supplementary diet did not negatively affect overall lamb carcass quality. This effect was similarly not unexpected, because pasture and a supplementary feed did not affect ADG, and generally, similar ADG leads to similar SLW, HCW, CCW, which is positively related to HCW [60]. Other recent studies have similarly reported pasture-grazing and a supplementary diet to not significantly affect sheep carcass attributes [10,61].

### 4.3. Meat Sensory Quality

Meat color, including L*, a*, and b*, affects consumer perception of meat quality [31,62,63]. Compared with stall-fed lambs, pasture-grazed lambs had lower L* and higher b* values. Accordingly, the color attributes of stall-fed lamb meat were more desirable. Because meat color is affected by fat deposition and oxidation [64], the lower L* value of lambs grazed on pasture likely reflects the lower fat content, and greater metabolic oxidation because of increased physical activity [65]. The lower b* value may indicate the higher IMF observed in stall-fed animals [66]. Ke et al. [9] similarly reported the longissimus muscle of pasture-fed lambs to have a lower L*, and comparable or lower a* and b* to stall-fed lambs. We also report the longissimus muscle of pasture-grazing lambs fed a supplement to have lower L* values and higher a* and b* values. Results for a* values may partly be because there are more water-soluble molecules with higher antioxidant activity (such as vitamin C) in fresh herbage that could enhance antioxidant effects in muscle of pasture-grazed lambs that were also fed a supplement. Lower phenolic compounds in concentrates given to stall-fed animals could also reduce myoglobin oxidation and meat discoloration [62].

Meat tenderness and juiciness are two characteristics that affect consumer acceptability and satisfaction [10,58]. Generally, meat tenderness correlates negatively with WBSF, and juiciness is strongly negative correlated with CL and DL [7,63]. We report the longissimus muscle of pasture-grazed lambs to be less tender (with a higher WBSF) and similarly juicy (with similar CL and DL) to stall-fed lambs. In a related publication in which meat tenderness and juiciness were examined in pasture-grazed and stall-fed lambs, the former was similarly less tender but comparably as juicy as the latter [47]. The increased tenderness of stall-fed lamb muscle could be a function of decreased fiber content, reduced strength of connective tissue, and disintegrating myofibrillar proteins and connective tissue [67,68]. However, Maiorano et al. [69] considered that a greater ADG in lamb meat might increase soluble collagen and lead to increased tenderness. Our analysis revealed that the longissimus muscle of pasture-grazed lambs fed a supplement was less tender (with a higher WBSF) and less juicy (with a higher DL) than stall-fed animals. These contradictions reflect inconsistencies in publications used for analysis. Other publications [10,31] have reported no difference in tenderness or juiciness of the longissimus muscle in pasture-grazed lambs fed a supplement and stall-fed animals. Zhao et al. [70] reported a higher WBSF (13.7%), but differences were not significant; lower meat juiciness was indicated by a significantly higher (6.23%) CL for pasture-grazed lambs fed a supplement compared with stall-fed animals. Therefore, based on available data, it remains uncertain if meat from pasture-grazed lambs fed a supplement is more or less tender or juicy than meat from stall-fed animals.

### 4.4. Meat Nutritional Quality

Meat nutritional quality attributes, including moisture, protein, IMF, and ash, greatly affect consumer acceptability and market consumption potential [7,71,72]. Our analysis revealed that the longissimus muscle of pasture-grazed lambs had higher protein and lower IMF and ash contents compared with stall-fed animals. These results may be because greater muscle activity and energy metabolism reduces fat deposition, and increased myoprotein formation and protein content [47,70]. The same was reported for stall-fed animals, where greater absorption of glucose, lactate, and propionate promoted fat deposition [73].

We report that pasture-grazed lambs fed a supplement have greater protein and lower IMF contents than stall-fed animals. These findings may be because pasture-fed lambs receive more concentrates, leading to similar energy intake but greater exercise activity (promoting the breakdown of carbohydrates and fats and promoting protein synthesis) than animals that have been solely stall-fed. IMF content correlates with flavor intensity and with the type of fatty acids that affect human health [7,74]. From a human-health perspective, high IMF contents in longissimus muscle are widely considered to be unhealthy, with a higher total fat intake contributing to increased obesity and coronary heart disease.

## 5. Conclusions

Compared with stall-fed lambs, pasture-grazed animals had lower average daily gain, slaughter live weight, hot carcass weight, cold carcass weight, and rib eye area, while pasture-grazed animals fed a supplementary feed had comparable growth performance and carcass attributes to stall-fed animals. In terms of meat quality, pasture-grazed lambs had reduced color, tenderness, and IMF attributes, but significantly higher protein content. Pasture-grazed lambs fed a supplement had improved color and protein attributes, but tenderness and intramuscular fat remained significantly lower than meat from stall-fed animals. Future research should investigate the influence of feeding systems on the reproductive capacities, flavor profile, and amino acid composition of lamb meat.

## Figures and Tables

**Table 1:** Terms and single cross-referencing used in the highly sensitive search for the retrieval of studies with sheep on different systems.

Acronym	Terms and Cross-References
Population (P)	(“Lamb” OR “Sheep” OR “*Ovine*” OR “Ram” OR “Wether” OR “Ewe”)
	AND
Intervention (I) and comparison (C)	(“Feeding method” OR “Feeding pattern” OR “Grazing” OR “Barn fattening” OR “Feeding regimens” OR “Rearing systems” OR “confinement” OR “feeding system”)
	AND
Outcome (O)	(“Animal performance” OR “Weight gain” OR “performance” OR “meat quality”)
	NOT
Study design (S)	(“Review” OR “Meta-analysis”)

**Table 2:** Summary of the characteristics of the 23 published papers included in the meta-analysis.

Author	No. of Comparisons	Study Design	Country	Species/Cultivar	N	Treatments	Evaluated Variables ^1^
[23]	2	RCT	Tunisia	Barbarine male lambs	28	Stall-feeding/grazing pasture plus supplementation	ADG; SLW; CCW; DP; protein; IMF; Ash
[24]	2	RCT	Italy	Sarda nursing ewes	48	Stall-feeding/grazing pasture plus supplementation	ADG; SLW
[25]	2	RCT	Italy	Alpagota, Brogna and Foza lambs	36	Stall-feeding/pasture-grazing	ADG; HCW; DP; L*; a*; b*
[26]	2	RCT	Spain	Talaverana-breed lambs	53	Stall-feeding/grazing pasture plus supplementation	ADG; SLW; CCW; DP; L*; a*; b*; CL; WHC
[27]	2	RCT	Brazil	Dorper x Santa Ynez crossbred lambs	72	Stall-feeding/grazing pasture plus supplementation	ADG; SLW
[10]	3	RCT	Brazil	Texel breed	30	Stall-feeding/pasture-grazing/grazing pasture plus supplementation	SLW; HCW; CCW; DP; REA; L*; a*; b*; WBSE; CL
[28]	3	RCT	Brazil	Dorper x Santa Ynez crossbred lambs	72	Stall-feeding/pasture-grazing/grazing pasture plus supplementation	ADG; SLW; HCW; CCW; DP; REA; DL
[29]	2	RCT	French	Limousine-breed lambs	307	Stall-feeding/pasture-grazing	ADG; SLW; CCW
[30]	2	RCT	Spain	Churra Tensina breed lambs	48	Stall-feeding/pasture-grazing	SLW; IMF
[31]	3	RCT	France	Romane lambs	36	Stall-feeding/pasture-grazing/grazing pasture plus supplementation	ADG; SLW; CCW; L*; a*; b*
[32]	2	RCT	Brazil	Cruzas Ile de France x Texel	12	Stall-feeding/pasture-grazing	ADG; HCW; CCW; DP; REA
[33]	3	RCT	Brazil	Ewe lambs	24	Stall-feeding/pasture -razing/grazing pasture plus supplementation	SLW; ADG; Moisture; CP; Ash; EE
[34]	2	RCT	Poland	Polish Merino ram lambs	49	Stall-feeding/pasture-grazing	ADG; SLW; CCW; REA
[35]	3	RCT	France	Romane lambs	36	Stall-feeding/pasture-grazing/grazing pasture plus supplementation	EE
[36]	2	RCT	Tunisia	Weaned male lambs	24	Stall-feeding/grazing pasture plus supplementation	ADG; SLW; HCW; CCW; DP; L*; a*; b*; IMF
[37]	3	RCT	Turkey	Dorper lambs	45	Stall-feeding/pasture-grazing/grazing pasture plus supplementation	ADG; SLW; HCW; CCW; DP; L*; a*; b*; WBSE; CL; DL
[38]	2	RCT	Turkey	Chios lambs	34	Stall-feeding/grazing pasture plus supplementation	L*; a*; b*; CL; Moisture; protein
[39]	3	RCT	Turkey	Tuj lambs	39	Stall-feeding/pasture-grazing/grazing pasture plus supplementation	ADG; SLW; HCW; CCW; DP; REA
[40]	2	RCT	Turkey	Kivircik male lambs	22	Stall-feeding/pasture-grazing	SLW; HCW; WBSF; L*; a*; b*; CL; DL
[8]	2	RCT	Turkey	Norduz lambs	30	Stall-feeding/pasture-grazing	L*; a*; b*; Moisture; CP; Ash; EE;
[41]	2	RCT	India	Malpura lambs	22	Stall-feeding/grazing pasture plus supplementation	ADG
[42]	3	RCT	Spain	Rasa Aragonesa lambs	94	Stall-feeding/pasture-grazing/grazing pasture plus supplementation	ADG; SLW; HCW; CCW; DP
[43]	3	RCT	Spain	Montesina lamb	30	Stall-feeding/pasture-grazing/grazing pasture plus supplementation	Moisture; CP; EE; Ash; L*; a*; b*; WHC
[44]	3	RCT	China	Tan lambs	50	Stall-feeding/pasture-grazing/grazing pasture plus supplementation	SLW; DP; REA; L*; a*; b*; CL; Moisture; protein; Ash
[12]	3	RCT	China	Tan lambs	50	Stall-feeding/pasture-grazing/grazing pasture plus supplementation	ADG; SLW; HCW; IMF
[45]	2	RCT	China	Mongolia lambs	20	Stall-feeding/pasture-grazing	SLW; HCW; L*; a*; b*; WBSE; Moisture; protein; IMF; Ash
[46]	2	RCT	China	Mongolia lambs	24	Stall-feeding/pasture-grazing	SLW; L*; a*; b*; WBSE; protein; IMF; Ash
[47]	2	RCT	China	Hulunbuir lambs	44	Stall-feeding/pasture-grazing	SLW; HCW; DP; L*; a*; b*; WBSE; CL; DL; WHC; Moisture; IMF
[48]	3	RCT	China	Gangba lambs	30	Stall-feeding/pasture-grazing/grazing pasture plus supplementation	DP; CL; DL; Moisture; CP; Ash; EE
[49]	2	RCT	China	Small Tail Han lambs	24	Stall-feeding/pasture-grazing	EE; L*; a*; b*

^1^ ADG, average daily gain; a*, redness; b*, yellowness; CCW, cold carcass weight; CL, cooking loss; DL, drip loss; DP, dressing percentage; HCW, hot carcass weight; IMF, intramuscular fat; L*, lightness; RCT, randomized controlled trial; REA, rib eye area; SLW, slaughter live weight; WBSF, Warner–Bratzler shear force; WHC, water-holding capacity.

**Table 3:** Descriptive statistics of the complete database used in the meta-analysis (stall-feeding vs. pasture-grazing).

Item	NC	Mean		Minimum		Maximum		SD	
		SF	PG	SF	PG	SF	PG	SF	PG
Growth rate									
ADG (g/d) ^1^	24	206.45	172.64	22.20	53.90	332.00	317.00	96.94	82.83
Carcass attributes									
SLW (kg)	34	33.78	30.30	10.80	10.70	48.60	46.30	9.33	7.98
HCW (kg)	24	15.64	13.89	9.96	9.70	23.56	17.26	4.13	2.48
CCW (kg)	20	15.65	13.73	10.70	8.66	22.10	21.20	3.50	3.68
DP (%)	20	45.91	44.50	40.42	37.50	51.06	51.37	3.44	3.84
REA (cm^2^)	12	12.75	10.68	8.96	8.85	14.70	12.60	2.18	1.65
Edible quality of longissimus muscle									
Lightness	24	38.00	36.43	23.01	25.21	44.80	41.37	6.90	5.16
Redness	24	14.03	14.35	6.60	6.70	22.56	22.46	6.18	5.47
Yellowness	24	8.27	9.05	1.62	0.76	14.17	13.49	3.93	3.66
WBSE (N)	12	34.08	40.87	5.80	7.11	55.55	71.28	19.29	20.69
Cooking loss (%)	12	25.30	26.31	14.73	14.75	36.59	39.71	9.35	11.34
Drip loss (%)	10	3.42	3.92	1.33	1.29	9.06	9.11	3.29	3.17
Nutritional quality of longissimus muscle									
Moisture (%)	12	72.82	73.94	68.93	71.76	75.90	76.17	2.36	1.53
Protein (%)	14	21.66	21.73	18.88	18.92	24.94	24.55	2.03	2.03
Intramuscular fat (%)	22	3.76	2.66	1.64	1.05	6.57	4.25	1.45	1.04
Ash (%)	14	1.05	1.01	0.87	0.84	1.21	1.25	0.12	0.15

^1^ ADG, average daily gain; CCW, cold carcass weight; DP, dressing percentage; HCW, hot carcass weight; PG, pasture-grazing; NC, no. of comparisons; REA, rib eye area; SD, standard deviation; SF, stall-feeding; SLW, slaughter live weight; WBSF, Warner–Bratzler shear force.

**Table 4:** Descriptive statistics of the complete database (stall-feeding vs. grazing pasture plus supplementation).

Item	NC	Mean		Minimum		Maximum		SD	
		SF	GPS	SF	GPS	SF	GPS	SF	GPS
Growth rate									
ADG (g/d) ^1^	36	187.78	177.08	22.20	17.80	332.00	299.00	91.07	86.43
Carcass attributes									
SLW (kg)	44	33.20	32.03	9.37	10.20	48.60	46.90	9.46	8.47
HCW (kg)	26	14.65	14.02	9.96	9.20	19.89	19.51	3.49	2.75
CCW (kg)	30	16.64	15.19	10.70	9.00	23.20	21.90	3.87	3.91
DP (%)	34	45.92	45.71	40.42	39.40	54.63	51.25	3.69	2.57
REA (cm^2^)	18	12.48	11.67	8.96	9.86	14.70	14.84	2.69	1.57
Edible quality of longissimus muscle									
Lightness	26	43.11	42.10	37.66	37.64	65.28	62.04	7.03	6.33
Redness	26	14.68	15.55	3.88	4.36	22.02	24.22	6.12	6.03
Yellowness	26	9.26	9.71	3.14	3.02	14.17	14.65	4.35	4.04
WBSE (N)	8	33.45	33.06	19.03	24.33	38.26	38.26	9.61	6.16
Cooking loss (%)	22	30.55	31.06	22.13	19.49	36.59	38.58	5.95	6.85
Drip loss (%)	6	4.77	4.22	1.61	2.00	9.06	6.58	3.85	2.29
Nutritional quality of longissimus muscle									
Moisture (%)	14	74.08	74.30	68.93	71.57	76.52	76.88	2.78	1.91
Protein (%)	18	33.56	34.69	18.88	19.51	76.70	80.80	24.20	25.54
Intramuscular fat (%)	20	6.56	5.44	1.64	1.93	20.30	17.00	6.96	5.41
Ash (%)	16	1.92	1.99	1.01	0.85	4.30	5.10	1.44	1.78

^1^ ADG, average daily gain; CCW, cold carcass weight; DP, dressing percentage; HCW, hot carcass weight; GPS, grazing pasture plus supplementation; NC, no. of comparisons; REA, rib eye area; SD, standard deviation; SF, stall-feeding; SLW, slaughter live weight; WBSF, Warner–Bratzler shear force.

**Table 5:** Effect of different feeding systems on the carcass attributes of lambs in terms of effect size, heterogeneity, and publication bias.

Feeding Regimen	Outcomes	No. of Comparisons	SMD (95% CI)		Heterogeneity		Publication Bias Egger
			Random Effect	*p*-Value	*I* ^2^	*p*-Value	
Pasture-grazing	SLW (kg) ^1^	17	−0.55 (−0.71, −0.39)	<0.001	88.9	<0.001	<0.001
	HCW (kg)	12	−0.28 (−0.53, −0.03)	0.031	93.6	<0.001	0.055
	CCW (kg)	10	−0.68 (−0.86, −0.49)	<0.001	91.8	<0.001	0.325
	DP (%)	10	0.02 (−0.24, 0.29)	0.879	89.7	<0.001	0.721
	REA (cm^2^)	6	−1.57 (−1.99, −1.14)	<0.001	75.7	0.001	0.707
Grazing pasture plus supplementation	SLW (kg)	22	0.06 (−0.15, 0.27)	0.588	91.7	<0.001	0.535
	HCW (kg)	13	0.08 (−0.20, 0.36)	0.578	92.4	<0.001	0.903
	CCW (kg)	15	−0.17 (−0.42, 0.07)	0.159	79.2	<0.001	0.001
	DP (%)	17	0.15 (−0.08, 0.39)	0.201	87.4	<0.001	0.711
	REA (cm^2^)	9	0.42 (−0.03, 0.86)	0.065	92.3	<0.001	0.348

^1^ CCW, cold carcass weight; DP, dressing percentage; HCW, hot carcass weight; SLW, slaughter live weight; SMD, standardized mean difference.

**Table 6:** Effect of different feeding systems on the lamb meat edible quality attributes in terms of effect size, heterogeneity, and publication bias.

Feeding Regimen	Outcomes	No. of Comparisons	SMD (95% CI) ^1^		Heterogeneity		Publication Bias Egger
			Random Effect	*p*-Value	*I* ^2^	*p*-Value	
Pasture-grazing	Lightness	12	−0.63 (−0.91, −0.35)	<0.001	93.7	<0.001	0.064
	Redness	12	−0.03 (−0.28, 0.21)	0.801	86.8	<0.001	0.451
	Yellowness	12	0.36 (0.09, 0.63)	0.009	93.0	<0.001	0.537
	Warner–Bratzler shear force (N)	6	1.13 (0.73, 1.52)	<0.001	91.9	<0.001	0.260
	Cooking loss (%)	6	0.51 (0.10, 0.91)	0.015	94.6	<0.001	0.133
	Drip loss (%)	5	0.06 (−0.32, 0.45)	0.751	92.7	<0.001	0.086
Grazing pasture plus supplementation	Lightness	13	−0.57 (−0.82, −0.32)	<0.001	74.3	<0.001	0.200
	Redness	13	0.70 (0.44, 0.96)	<0.001	78.6	<0.001	0.051
	Yellowness	13	0.47 (0.21, 0.74)	<0.001	87.7	<0.001	1.000
	Warner–Bratzler shear force (N)	4	0.44 (−0.12, 1.00)	<0.001	89.1	<0.001	0.089
	Cooking loss (%)	11	0.74 (0.44, 1.03)	<0.001	88.7	<0.001	0.533
	Drip loss (%)	3	0.49 (−0.11, 1.10)	0.111	95.2	<0.001	0.296

^1^ CI, confidence interval; SMD, standardized mean difference.

**Table 7:** Effect of different feeding systems on the lamb meat nutritional quality attributes in terms of effect size, heterogeneity, and publication bias.

Feeding Regimen	Outcomes	No. of Comparisons	SMD (95% CI) ^1^		Heterogeneity		Publication Bias Egger
			Random Effect	*p*-Value	*I* ^2^	*p*-Value	
Pasture-grazing	Moisture (%)	6	0.37 (0.03, 0.71)	0.031	0.00	0.478	0.060
	Protein (%)	7	0.17 (−0.21, 0.55)	0.392	92.6	<0.001	0.548
	IMF (%)	11	−1.06 (−1.34, −0.78)	<0.001	92.0	<0.001	<0.001
	Ash (%)	7	−0.80 (−1.15, −0.46)	<0.001	67.8	0.005	1.000
Grazing pasture plus supplementation	Moisture (%)	7	−0.40 (−0.74, −0.06)	0.021	76.4	<0.001	0.035
	Protein (%)	9	0.72 (0.38, 1.06)	<0.001	86.3	<0.001	0.009
	IMF (%)	7	−0.88 (−1.22, −0.54)	<0.001	84.8	<0.001	0.012
	Ash (%)	8	−0.77 (−1.15, −0.39)	<0.001	85.8	<0.001	0.711

^1^ CI, confidence interval; IMF, intramuscular fat; SMD, standardized mean difference.

## Data Availability

The datasets used and analyzed during the current study are available from the corresponding author upon reasonable request.
